# Exploration of Factor Structure and Measurement Invariance by Gender for a Modified Shortened Adapted Social Capital Assessment Tool in India

**DOI:** 10.3389/fpsyg.2019.02641

**Published:** 2019-12-11

**Authors:** Md Zabir Hasan, Jeannie-Marie Leoutsakos, William T. Story, Lorraine T. Dean, Krishna D. Rao, Shivam Gupta

**Affiliations:** ^1^Department of International Health, Johns Hopkins Bloomberg School of Public Health, Baltimore, MD, United States; ^2^School of Medicine, Johns Hopkins University, Baltimore, MD, United States; ^3^Department of Community and Behavioral Health, The University of Iowa, Iowa City, IA, United States; ^4^Department of Epidemiology, Johns Hopkins Bloomberg School of Public Health, Baltimore, MD, United States

**Keywords:** social capital, gender, measurement invariance, factor analysis, India

## Abstract

Social capital is defined as the nature of the social relationship between individuals or groups and the embedded resources available through their social network. It is considered as a critical determinant of health and well-being. Thus, it is essential to assess the performance of any tool when meaningfully comparing social capital between specific groups. Using measurement invariance (MI) analysis, this paper explored the factor structure of the social capital of men and women measured by a modified Shortened Adapted Social Capital Assessment Tool (SASCAT-I) in rural Uttar Pradesh (UP), India. The study sample comprised 5,287 men (18–101 years) and 7,186 women (15–45 years) from 6,218 randomly selected households who responded to SASCAT-I during a community-level cross-sectional survey. Social capital factor structure was examined by both exploratory and confirmatory factor analysis (CFA), and MI across genders was investigated using multigroup CFA. While disregarding gender, four unique factors (*Organizational Participation*, *Social Support*, *Trust*, and *Social Cohesion*) represented the structure of social capital. The MI analysis presented a partial metric-invariance indicating factor loadings for *Organizational Participation* and *Social Support* were the same across genders. The gender-stratified analysis demonstrated that a four-factor solution was best fitted for both men and women. Men and women of rural UP interpreted social capital differently as the perception of *Trust* and *Social Cohesion* varied across genders. For any future applications of SASCAT-I, we recommend gender-stratified factor analysis to quantify social capital’s measure, acknowledging its multidimensionality.

## Introduction

Social capital has become one of the most contested concepts in the social sciences over the last few decades since Robert Putnam’s groundbreaking work on democracy and civic engagement (Putnam, 1995). While in economics (Loury, 1992; Knack and Keefer, 1997) and political science (Putnam, 2000), the concept of social capital was debated from much earlier, research on social capital gained its traction in health systems and policy in early 2000 (Kawachi et al., 2008). The role of social capital in global health became more imperative in 2010 after the World Health Organization’s Commission for Social Determinants of Health acknowledged social capital as a crosscutting determinant of health and inequity (World Health Organization [WHO], 2010).

Despite its theoretical use in different fields of knowledge, there is no universal definition of social capital yet. Sociologist Pierre Bourdieu first gave a concise definition of social capital, “…*the aggregate of the actual or potential resources which are linked to possession of a durable network*… *[it] provides each of its members with the backing of the collectively owned capital*” (Bourdieu, 1986). Bourdieu’s definition focuses on the socio-structural aspect, which identifies social capital as an individual’s attribute. However, its utility can go beyond the individual level to group, community, and even broader social space (nation!). Robert Putnam focused on this communal attribute and identified social capital as characteristics (such as trust, civic engagement, cohesion, reciprocity, etc.) of any social organization that facilitates coordinated action (Putnam, 1993, 1995). Based on the same philosophical standpoint, American sociologist James Coleman theorized social capital as a multifaceted construct. According to him,

“*It is not a single entity*… *with two elements in common: they all consist of some aspect of social structures, and they facilitate certain actions of actors*… *social capital inheres in the structure of relations between actors and among actors*” (Coleman, 1988).

This required a multidimensional conceptualization of social capital to understand how it affects our everyday life. Social capital is generally classified into structural and cognitive components. Structural social capital characterizes individuals and their social network, indicating “*objective measures of what people do*” (Islam et al., 2006). It features characteristics such as group membership, social support, collaboration, and political engagement. Cognitive social capital symbolizes how people “*feel*” as reflected by social norms, trust in the community, belongingness, and reciprocity (De Silva and Harpham, 2007). Szreter and Woolcock (2004) proposed bonding and bridging as two additional dimensions of social capital based on group composition. Bonding social capital develops among individuals of a group who have a similar social identity (Islam et al., 2006). Stronger bonding social capital encourages stronger group identity and enforcement of social norms. On the other hand, bridging social capital represents the relationship between individuals with different social characteristics (such as class, religion, and ethnicity) or within the hierarchical network of individuals with a differential level of resources and power (Varshney, 2003; Szreter and Woolcock, 2004). Bridging social capital is particularly important in relation to access to financial resources, information, or healthcare-seeking as people often need to go beyond their imminent social network to gain access to these resources.

Despite the lack of a universal definition or classification due to conceptual ambiguity, social capital plays a critical role in everyday life. And this happens in five pathways. First, social capital allows the dissemination of information through the network of social links. Second, beyond information, other instrumental resources can be accessed (such as borrowing money) through reciprocity or mutual-aid within the social network. Similarly, being a part of any specific social network gives power and exclusivity to people. An example can be being a part of a credit union gives its member access to resources and exclude the outsiders (Kawachi et al., 2008). This is the third pathway. The last two pathways are related to how social capital works at the group level. Communities with higher social capital generally show greater social integration and higher participation in voluntary organizations by their members (Chwe, 1999). Notably, in the time of disaster preparedness, relief, and recovery, we can see communities with higher social capital naturally coming together (Aida et al., 2013). Last, the emergence of social capital is also linked with social identity, cohesiveness, and conformation to the existing social norms. This phenomenon can affect the social environment and human health. A neighborhood with strong solidarity can impose informal social control to prevent violent crime, vandalism, or littering in the street (Takagi, 2013). On the other hand, this type of social control can be coercive as it also enforces social categorization and ethnic and gender stereotyping.

### Social Capital in Uttar Pradesh, India, and Its Relationship With Gender

Our study is based in Uttar Pradesh (UP), a northern state of India, which is the home of 220 million people. Similar to the other parts of India, UP is currently experiencing exponential growth in the economy and information technology (USAID and K4Health, 2010). However, while India has demonstrated substantial achievement in increasing life expectancy and improving literacy and quality of life, considerable disparities exist in UP based on geographical, gender, socioeconomic, and educational factors (Reddy et al., 2011). Although previous studies measured social capital in other states of India, such as Andhra Pradesh, Nagaland, and Tamil Nadu (De Silva and Harpham, 2007; Kaplan et al., 2018; Palanisamy et al., 2018), only a few studies explored social capital in the context of rural UP.

Historically, caste and social hierarchy have been entrenched in everyday individual and community life in India, more specifically in UP (Kowal and Afshar, 2015). Power, gender dynamics, and socioeconomic composition often led to the exclusion of women, lower castes, and religious minorities from decision making in the rural community (Kumar et al., 2016; Scott et al., 2017). While investigating the role of gram panchayat (GP) as a grassroots-level democratic institution, Sudha Pai explored the implication of the deep-seated social segmentation on the social capital of rural UP. She highlighted the contextual effect of gender divisions in the rural society of UP, which determines the development of social capital within and between groups (Pai, 2001). Other studies also observed a substantial difference in civic engagement and group participation–as a proxy of social capital–due to gender, caste, and class differences (Lise, 2000; Mayer, 2001; Hans, 2014). Thus, in the context of northern India–specifically in UP–gender plays a critical role in how social capital is developed.

Social capital has been shown to vary by gender due to differences in composition and participation of men and women in their social networks. In UP, this divergence of social participation based on gender is more extreme. A qualitative study by Hebert et al. (2019) showed that the prevailing societal norm on UP restricts women’s movement outside the household, limiting their economic activity and educational endeavor. It is expected for women to get married and live their life within the “domestic sphere.” With respect to structural social capital, social networks of women are more likely to feature kin and neighbors, while men’s networks are more likely to feature friends, coworkers, and advisers (Moore, 1990; Burt, 1998; Kim, 2014). Even though men and women have a similar number of organizational memberships, Chua et al. (2016) observed that traditionally, women were more affiliated with domestic life, religion, and community affairs. On the other hand, men’s organizational membership tends to be more economically oriented (O’Neill and Gidengil, 2013; Chua et al., 2016). Thus, the prevailing societal norm of UP limits women’s access to resources beyond their limited social network and restricting the development of social capital.

The difference in cognitive social capital across genders is more nuanced and based on culture. Traditionally, the patriarchal nature of Indian society–specifically Northern India–reinforced an inegalitarian culture for women (Rajadhyaksha and Velgach, 2015). From childhood to adulthood, men and women were often socialized into the prescribed categories of masculinity and femininity (Chua et al., 2016). Exploring the social role of women, Ridgeway (2011) explained that women are generally perceived to be caring and communal, and men as more agentic and strategic. These social roles may affect how men and women perceived the cognitive aspects of social capital, such as trust and cohesiveness (Cross and Markus, 1993). However, the literature is limited when it comes to exploring the difference in trust and cohesiveness across genders in the context of UP.

### Measurement of Individual Social Capital Across Genders

Social capital is a multidimensional concept with multiple constructs for both structural and cognitive components. So a better measurement of social capital should include a battery of indicators that enumerate not only the span of one’s social network and the embedded resources that he/she has access to but also the quality of the social relationships (such as trust, shared value, and social norm). This makes a quantitative measurement of social capital particularly difficult because unlike the structural component (such as social participation or social support), cognitive social capital (trust, social cohesion, etc.) cannot be objectively measured (Carrillo Álvarez and Riera Romaní, 2017). Thus, the factor analytical model is often used to measure social capital, considering it as a latent variable (Chen et al., 2009; Story, 2014; Kaplan et al., 2018).

In the health and development literature, social capital is mostly considered as an explanatory variable and researchers explore its association with a host of outcome variables including life satisfaction, well-being, economic activity, or health (Locher et al., 2005; Westermann et al., 2005; Kavanagh et al., 2006; Chuang and Chuang, 2008; Kim, 2014; Leeves and Herbert, 2014; Chua et al., 2016; Lu et al., 2018). Specifically, in the context of community-based multi-item surveys, a relatively short measurement instrument for social capital is often preferred (Harpham et al., 2002; Uphoff et al., 2013; Agampodi et al., 2015).

While multi-item tools are used to measure social capital, often researchers assume that the meaning of the latent factors is the same across multiple groups. To validate this assumption, it is necessary to assess that there is no association between the item response or the latent factor with the characteristics of the respondent (Mellenbergh, 1989). Given the importance of social capital as a critical social determinant of health (World Health Organization [WHO], 2010), its potential variation across genders due to differential measurements is still an unanswered question. Any difference in the performance of a tool to measure social capital across genders may have a multifaceted impact on research by affecting several critical decisions, including whom to include as respondents, when to collect data, who collects the data, and how to analyze the data (Morgan et al., 2016). All these may bias the finding of health systems research, leading to gender inequality in health policy and interventions (Millsap and Kwok, 2004; Guenole and Brown, 2014). While a few studies explore the performance of social capital measurement tools across other social stratifiers (Davidov and Coromina, 2013; Gesthuizen et al., 2013; Liu et al., 2018), there is little evidence of how the quantitative tools used in these studies performed when measuring social capital across genders. In addition, these studies mostly included only one construct of social capital as a proxy measure. This study aims to examine how a short and simple quantitative tool performs during the measurement of social capital across genders in rural India.

In this study, we have assessed social capital using a modified shortened adapted social capital assessment tool (SASCAT), which will be addressed as SASCAT-India (or SASCAT-I in short). De Silva et al. (2007) initially developed SASCAT specifically for India and implemented the tool in the Young Lives Research Project on childhood poverty after performing psychometric and cognitive validation (Silva et al., 2006). Since then, multiple psychometric assessments and validation studies on SASCAT were performed in the context of India and South Asia (Silva et al., 2006; De Silva and Harpham, 2007; De Silva et al., 2007; Story et al., 2015; Kaplan et al., 2018). The original SASCAT presented three constructs (Group membership/Social support, Citizenship, and an overall Cognitive social capital). However, cognitive validation of SASCAT in Bangladesh considered three structural (Group membership, Social support, and Collective action) and two cognitive (Trust and Social cohesion) constructs (Story et al., 2015).

The objective of this study is to explore the factor structure of social capital for men and women. To achieve this objective, we used the factor analytic framework to explore the factor structure of social capital and measurement invariance (MI) across genders as measured by the SASCAT-I in rural UP, India.

## Materials and Methods

### Data Source

The analysis was based on the baseline household survey for a multisectoral rural development initiative by HCL Foundation (2018) known as Project Samuday. The survey was conducted from June to August 2017 in two rural districts of UP, Hardoi and Sitapur. Adjacent to Lucknow, the capital of UP, both districts are considered to be rural and performing poorly on critical demographic, economic, and health indicators (International Institute for Population Sciences and ICF, 2017).

Adopting a multistaged cross-sectional design, the survey was conducted among 6,218 randomly selected households from 346 GPs. Each GP consists of one to four villages and is constitutionally accredited as the rural governing body (Ministry of Panchayati Raj and Government of India, 2017). From each GP, the sampling frame was developed using the service area of the accredited social health activist (ASHA), the assigned community health worker from the Government of India. In general, one ASHA is assigned to serve a thousand population of the GP (Government of India, 2018). The survey randomly selected the service area of one ASHA as the primary sampling unit (PSU), and 17–18 households were randomly selected for interviews from each PSU.

Ethical approval for the study was received from the Institutional Review Board Office of Johns Hopkins Bloomberg School of Public Health and locally from the Center for Media Studies, New Delhi, India. In the study area, around 50% of women and 70% of men were literate (International Institute for Population Sciences and ICF, 2017). Thus, the survey did not receive any written informed consent from the participants considering the feasibility of the process. Following the standard research practice in India (International Institute for Population Sciences and ICF, 2014), before starting the interview, oral informed consent was received from the participants (adult or non-adult) and from the parents of the non-adult participants by trained data collectors. From each selected household, data collectors interviewed the household head (18–101 years) and all women between the ages of 15 and 49. The survey instrument included information related to social capital and sociodemographic characteristics. From each PSU, on average, 15 men and 20.8 women responded to SASCAT-I. The analytic sample of the study included 5,287 men (85% of the household heads) and 7,186 women with a response rate of over 99%.

### Measures

While implementing this tool in the context of UP, initial social capital questions were developed in English from SASCAT (Silva et al., 2006) and SASCAT-Bangladesh (Story et al., 2015). To translate the questions into Hindi, a bilingual panel of researchers performed two rounds of rapid cognitive interviews and incorporated appropriate local colloquialism, idioms, and vernacular terms (Beatty and Willis, 2007; Haeger et al., 2012). The final and contextually modified SASCAT-I was back-translated into English to check the translational validity of the questions. Table 1 summarizes the questions from SASCAT-I (see Supplementary Material for the complete tool).

**TABLE 1:** Social capital Questions from contextually modified Shortened and Adapted Social Capital Assessment Tool in India (SASCAT-I).

**Theoretically unique social capital constructs**	**Structure of shortened and adapted social capital assessment tool in India (SASCAT-I)**		
	
	**Question name**	**Description of the question**	**Type of response**
**Structural social capital indicators**			
Organizational participation with the community	Group participation	Number of community group you have participated in the last 12 months	Continuous
	Group benefit	Number of benefits received you have from the community groups in the last 12 months	Continuous
	Collective action	Worked together with other community members and attempted to address a problem or common issue of the village in the last 12 months	Binary
	Development discussion	Number of people you have spoken with about the development of the village in the past 12 months	Continuous
Social support	Emotional support	Number of emotional supports received from the community in the last 12 months	Continuous
	Financial support	Number of financial supports received from the community in the last 12 months	Continuous
	Informational support	Number of informational supports received from the community in the last 12 months	Continuous
**Cognitive social capital questions**			
Trust	Trust in leaders	Overall, trust in village leaders	Categorical
	Trust in strangers	Overall, trust in unfamiliar people residing in the village	Categorical
	Trust in neighbors	Overall, trust in village neighbors	Categorical
Social cohesion	Social harmony	People in this village generally have good relationships with each other	Categorical
	Sense of belonging	Feel that you belong to this village	Categorical
	Sense of fairness	People in this village will try to take advantage of you if they get the chance	Categorical

*All structural social capital indicators are recoded into binary indicators. Group participation and group membership are merged into one indicator called group membership due to high collinearity. All cognitive social capital indicators are framed as 3-point Likert responses (Yes = 3, Sometime = 2, and No = 1). Sense of Fairness is reversely coded (Yes = 1, Sometime = 2, and No = 3).*

Theoretically, SASCAT-I embodies four unique constructs of social capital, which include engagement with the community, social support, trust, and social cohesion. Structurally, the first seven questions of the tool measured an individual’s engagement with community and access to resources through social support, reflecting the level of structural social capital. These included two questions for engagement in formal or informal community groups, two for collective action with the community, and three for acquired social support (Table 1). The questions regarding engagement with community groups included: (1) *In the last 12 months, have you been a member of any of the following groups?* (Group Participation) and (2) *In the last 12 months, how have you participated in or benefited from the group?* (Group Benefit). Participation in any collective action with the community was elicited by (1) *In the last 12 months, have you worked together with other community members and attempted to address a problem or common issue of the village?* (Collective Action) and (2) *In the past 12 months, have you spoken with anyone about the development of your village?* (Development Discussion). Three separate questions were used to understand the number of sources from where emotional, financial, and informational social supports were acquired within the last 12 months (Emotional Support, Financial Support, Informational Support).

The last six questions of SASCAT-I explored two constructs of cognitive social capital, trust, and social cohesion using 3-point Likert scale type responses—yes, sometimes, or no. These questions were focused on measuring the attitudes and beliefs of an individual toward the community where he/she belongs. Among them, three questions are related to trust, which include trust in leaders, trust in strangers (any unfamiliar people residing in the village), and trust in neighbors. The last three questions of the tool measured social cohesion by asking the respondents: (1) *Do you think the majority of people in this village generally have good relationships with each other?* (Social Harmony), (2) *Do you feel that you have a sense of belonging to this village?* (Sense of Belonging), and (3) *Do you think that the majority of people in your village would try to take advantage of you if they got the chance?* (Sense of Fairness).

### Analytic Strategy

Data management and descriptive analysis were conducted using Stata version 15 (StataCorp, 2017), and factor analysis was performed using Mplus version 8.1 (Muthén and Muthén, 2017). First, descriptive analysis of the respondent’s characteristics and the social capital responses were explored, which was followed by an exploration of the bivariate association of social capital and gender using the chi-square test. Next, necessary modifications were made to transform the social capital responses into items for factor analysis.

Prior to focusing the analysis on MI, a well-fitting factor structure should be established. To do that, the sample was divided randomly into two equal subsets having an equal distribution of both genders. Exploratory factor analysis (EFA) was implemented using the first random subset (*n* = 6,207) to identify a tenable factor structure, which also has the necessary goodness of fit indices. Next, using the second subset (*n* = 6,266), a confirmatory factor analysis (CFA) was implemented to assess the generalizability of the possible factor structure (Huang and Cornell, 2015) to confirm if social capital measured by SASCAT-I conformed the theoretical constructs of social capital (Stafford et al., 2008; Elgar et al., 2011).

To assess MI of social capital factor structure across genders, multiple-group CFA was used (Chavez et al., 2018; Kim and Kamphaus, 2018). MI (also known as factorial invariance analysis) is used to quantitatively assess if the factor structures of latent variables (such as social capital) are the same across groups of the population. We evaluated MI across genders using multiple-group CFA as suggested by Gregorich (2007).

Measurement invariance analysis of the factor structure of social capital requires exploration of four types of hierarchical factorial invariance across the sample of men and women: configural, metric (also known as the pattern), scalar, and uniqueness factorial invariance. First, the least stringent invariance test, configural invariance, was implemented to understand if the latent factors of social capital were the same across groups (in this case, men and women). Non-invariance, at this level, means that one or more items loaded on a different factor social capital across the group. If configural invariance was achieved, equality constraints on the factor loadings of corresponding items are imposed to perform metric invariance analysis. This would indicate that each item had the same relationships with the latent construct across genders or contributes similarly.

If implementing the equality constraints significantly reduced the overall model fit compared to the configural model, it would indicate that one or more items loading were non-invariant and were not similar across men and women. In that case, modification indices were explored to identify the source of non-invariance, and equality constraints of loadings with the highest modification indices were sequentially released until a partial metric invariant model was achieved (Yoon and Kim, 2014). Moving forward, if a full metric model was supported, scaler invariance was assessed by imposing equality constraints on item intercepts across two groups. In case a partial metric model was supported in the previous stage, then intercepts of the released loading are left to be unconstrained.

The overall model fit was compared with the metric model, and a significantly worse model fit for the scalar invariance model would indicate that at least one item intercept was not the same among men and women. If a full scalar invariance model was not supported, then item intercepts were sequentially released until a partial scalar invariance was achieved or the invariance test was discontinued assuming that the social capital constructs were non-invariant across genders. In the final step, if a scalar invariance was supported, residual invariance was assessed by considering the residuals or the error terms of social capital items to be the same across genders. Based on the result of the MI analysis, social capital factor structures for men and women were separately reestimated using CFA. All factor analytical models were estimated using “weighted least square mean and variance” (WLSMV) adjusted estimator using a polychoric correlation matrix and holding factor variances fixed to one.

The overall fit of the models to the data was evaluated using multiple fit statistics. For any CFA model (including the configural invariance model), an adequate fit was considered if both comparative fit index (CFI) and Tucker-Lewis index (TLI) were ≥0.90, standardized root mean square residual (SRMR) was <0.08, and root mean square error of approximation (RMSEA) was <0.07 (Cheung and Rensvold, 2002; Hooper et al., 2008; Prudon, 2015). The fit of any nested model (metric, scalar, and residual invariance models) was evaluated, comparing the change in the goodness of fit after including the equality constraints. Any nested model was considered to have a better fit by achieving a non-significant Satorra-Bentler scaled chi-square difference test (Asparouhov and Muthen, 2007, 2019). Furthermore, for testing the fit of metric invariance, a change of ≥−0.01 CFI, ≥0.015 RMSEA, and ≥0.03 SRMR, and for scalar and residual invariance, ≥−0.01 CFI, ≥0.015 RMSEA, and ≥0.01 SRMR were considered indications of non-invariance (Chen, 2007). In addition to the goodness of fit indices, we considered the theoretical underpinning of social capital and parsimony to develop interpretable factor structures during the model selection process (Myung, 2000; Gregorich, 2007).

## Results

Table 2 presents the demographic characteristics of the respondents disaggregated by gender. In the analytical sample (*n* = 12,473)–compared to women–men were older (average age: men = 44 years, women = 30 years; *p*-value < 0.01). Men had significantly higher educational attainment and were more engaged in economic activities. Within the sample, 78.7%, (*n* = 9,816) respondents were married. Among the group who were never married, 93% (*n* = 1,876) of them were women. The majority of the participants were Hindu and belonged to the schedule caste and schedule tribe.

**TABLE 2:** Demographic characteristics of study participants disaggregated by gender.

**Participants characteristics**	**Men (*n* = 5,287)**		**Women (*n* = 7,186)**		***P*-values**	**Total (*n* = 12,473)**

Age (Mean)	44		30		0.00	36

	**n**	**%**	**n**	**%**		**n**
**Education**						
Illiterate	1,747	36	3,168	64	0.00	4,915
Up to primary	1,385	49	1,465	51		2,850
Above primary	2,155	46	2,545	54		4,700
**Occupation**						
Cultivator	2,878	96	133	4	0.00	3,011
Wage laborer	1,541	90	171	10		1,712
Other occupations	647	38	1,051	62		1,698
Unemployed/student/housewife	221	4	5,827	96		6,048
**Marital status**						
Single	136	7	1,876	93	0.00	2,012
Married	4,857	49	4,959	51		9,816
Widowed/divorced/separated	294	46	350	54		644
**Religion**						
Hindu	4,747	43	6,318	57	0.00	11,065
Muslim and others	540	38	868	62		1,408
**Social caste**						
General	915	41	1,344	59	0.00	2,259
Schedule caste and schedule tribe	2,509	44	3,185	56		5,694
Other backward caste and others	1,863	41	2,657	59		4,520

*% column represents row percentage of categories of the sample.*

**FIGURE 1:**
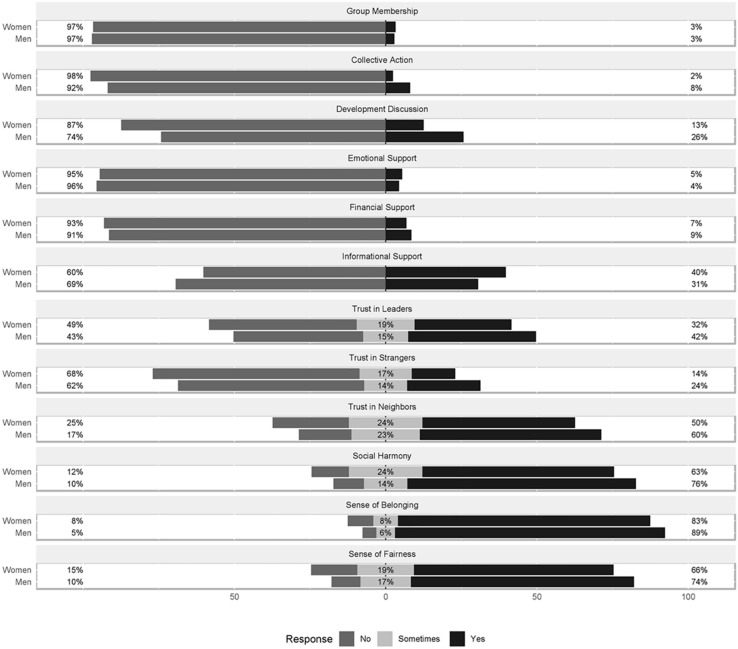
Percentage distribution of the 12 social capital indicators disaggregated by gender. All social capital indicators presented a statistically significant difference between men and women (χ^2^ *P* value < 0.05), except for group membership.

Table 3 presents the distribution of responses to the social capital indicators derived from SASCAT-I. The responses of the first two questions (group participation and group benefit) were highly correlated (correlation coefficient 0.98). Thus, we have developed one single indicator called “group membership” by merging those two indicators. We observed very low positive responses for the structural indicators compared to the six cognitive items. Thus, the six structural social capital items were recategorized into binary (yes/no) responses for analysis. Figure 1 shows the percentage distribution of the 12 social capital indicators disaggregated by gender.

**TABLE 3:** The response of the participants on the shortened and adapted social capital assessment tool in India (SASCAT-I).

**Indicator name**	**Type of response**	**Total (*n* = 12,473)**				**Men (*n* = 5,287)**				**Women (*n* = 7,186)**			
		**Mean**	***SD***	**Min**	**Max**	**Mean**	***SD***	**Min**	**Max**	**Mean**	***SD***	**Min**	**Max**
Group membership	Count	0.11	0.78	0	22	0.11	0.83	0	22	0.11	0.75	0	18
Collective action	Binary	0.05	0.21	0	1	0.08	0.27	0	1	0.02	0.15	0	1
Development discussion	Count	0.25	0.65	0	7	0.37	0.77	0	7	0.17	0.52	0	7
Emotional support	Count	0.10	0.55	0	9	0.10	0.56	0	9	0.11	0.53	0	7
Financial support	Count	0.09	0.35	0	5	0.11	0.38	0	4	0.08	0.32	0	5
Informational support	Count	0.46	0.72	0	7	0.44	0.79	0	7	0.47	0.66	0	6
Trust in leaders	3-point likert	1.90	0.90	1	3	1.99	0.92	1	3	1.83	0.88	1	3
Trust in strangers	3-point likert	1.53	0.79	1	3	1.63	0.85	1	3	1.46	0.73	1	3
Trust in neighbors	3-point likert	2.33	0.81	1	3	2.43	0.77	1	3	2.25	0.83	1	3
Social harmony	3-point likert	2.57	0.69	1	3	2.65	0.65	1	3	2.51	0.71	1	3
Sense of belonging	3-point likert	2.79	0.55	1	3	2.85	0.47	1	3	2.75	0.60	1	3
Sense of fairness	3-point likert	2.56	0.71	1	3	2.64	0.65	1	3	2.51	0.75	1	3

*Group participation and group membership are merged into one indicator called Group Membership due to high collinearity.*

Less than 10% of respondents reported any group membership, collective action, or receiving any emotional or financial support within the last 12 months. Men reported higher collective action and development discussion and acquiring financial support compared to women. On the other hand, the positive response (yes) for the six cognitive items ranged from 18.58% (trust in strangers, *n* = 2,318) to 85.89% (sense of belonging, *n* = 10,713). Men had a significantly higher positive response (yes), and women reported “sometimes” more frequently for all of these items. All indicators presented a statistically significant difference between men and women (*p*-value < 0.05), except for group membership.

Results for factor and MI analysis are presented in the following sequence: (a) Identifying a well-fitted factor structure of social capital, (b) assessment of MI of the well-fitted factor structure, and (c) based on the result of the MI reestimate the factor structure separately for men and women.

### Identifying a Well-Fitted Factor Structure of Social Capital

**FIGURE 2:**
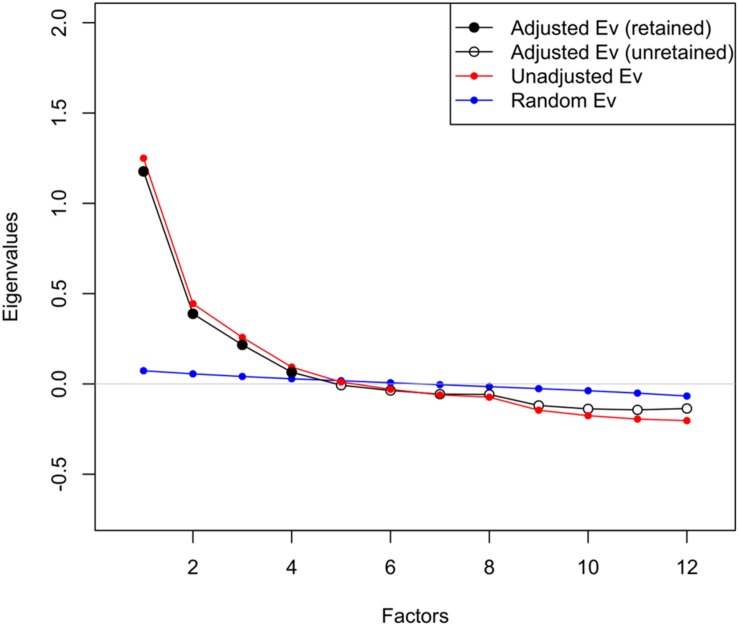
Scree plots of parallel analysis indicating the four possible factors emerged from the first random subset sample (*n* = 6,207).

To identify the best-fitted factor structure of social capital, we considered the theoretical perspective that was used to develop the original SASCAT, as well as an exploratory factor analytical approach. Theoretically, SASCAT-I embodied four constructs of social capital (engagement with the community, social support acquired from the community, level of trust, and social cohesion). We also found that a unique four-factor structure could be extracted from the data based on the result of parallel analysis (Figure 2).

Thus, we implemented a four-factor EFA model. The four-factor EFA model presented good model fit (RMSEA = 0.015, CFI = 0.994, TLI = 0.984, SRMR = 0.024, χ^2^ value = 57.829, df = 24, *p* < 0.01). Based on the geomin rotated factor loadings of the EFA model (Table 4), we identified the four possible factors and assigned their names–Factor 1: *Organizational Participation* (group membership, collective action, development discussion); Factor 2: *Social Support* (emotional support, financial support, and informational support); Factor 3: *Trust* (trust in leaders, trust in neighbors, and trust in strangers); and Factor 4: *Social Cohesion* (social harmony, sense of belonging, and sense of fairness).

**TABLE 4:** Factor loadings for exploratory and confirmatory factor analysis with four-factor solutions of 12 SASCAT-I indicators.

**Indicators**	**Unstandardized loadings for EFA^a^**				**Standardized loadings for CFA^b^**			
		
	**Factor 1**	**Factor 2**	**Factor 3**	**Factor 4**	**Factor 1**	**Factor 2**	**Factor 3**	**Factor 4**
Group membership	0.34^∗^	0.18^∗^	0.01	0.03	0.32^∗^			
Collective action	1.05^∗^	–0.01	−0.03^∗^	0.02	0.84^∗^			
Development discussion	0.53^∗^	0.19^∗^	0.09^∗^	–0.06	0.74^∗^			
Emotional support	0.01	0.54^∗^	0.10^∗^	−0.10^∗^		0.48^∗^		
Financial support	0.01	0.42^∗^	–0.03	0.08		0.42^∗^		
Informational support	0.01	0.48^∗^	−0.01^∗^	0.11^∗^		0.56^∗^		
Trust in leaders	0.01	0.04	0.73^∗^	0.03			0.68^∗^	
Trust in strangers	0.11^∗^	–0.04	0.46^∗^	0.01			0.52^∗^	
Trust in neighbors	–0.04	–0.01	0.54^∗^	0.36^∗^			0.74^∗^	
Social harmony	0.15^∗^	−0.09^∗^	0.11^∗^	0.66^∗^				0.72^∗^
Sense of belonging	−0.07^∗^	0.09^∗^	0.00	0.79^∗^				0.70^∗^
Sense of fairness	0.13^∗^	0.07	–0.04	0.39^∗^				0.36^∗^

*^*a*^EFA of random subset 1 (*n* = 6,207). ^*b*^CFA of Random subset 2 (n = 6,266). Goodness of fit indices for EFA: RMSEA = 0.015, CFI = 0.994, TLI = 0.984, SRMR = 0.024, χ^2^ value = 57.829, df = 24, *p* < 0.01. Goodness of fit indices for CFA: RMSEA = 0.027, CFI = 0.964, TLI = 0.95, SRMR = 0.042, χ^2^value = 273.44, df = 48, *p* < 0.01, ^∗^*P* < 0.05. EFA = exploratory factor analysis; CFA = confirmatory factor analysis; RMSEA = root mean square error of approximation; CFI = comparative fit index; TLI = Tucker-Lewis index; SRMR = standardized root mean square residual.*

Next, assuming the same four-factor solution as the latent structure of social capital, a CFA model was implemented to assess its generalizability. The CFA model also presented a good model fit (RMSEA = 0.027, CFI = 0.964, TLI = 0.95, SRMR = 0.042, χ^2^value = 273.44, df = 48, *p* < 0.01), and all 12-factor loadings were significantly different from 0 and higher than 0.3 (Sellin and Keeves, 1994). Thus, the factor analysis confirmed that a four-factor solution was the best-fitting factor structure for social capital.

### Assessment of MI of the Well-Fitted Factor Structure

Moving forward, we used the four-factor solution to assess whether social capital factor structure was quantitatively equivalent across genders. The model fit and comparison statistics are presented in Table 5. The four-factor configural invariance model (M1) presented a good fit to the data (RMSEA = 0.03, CFI = 0.95, TLI = 0.93, SRMR = 0.06, Δχ^2^ = 661.78, Δdf = 96, *p* < 0.05). This indicated that the composition of the factors was equal across genders (same items were associated with the same unique factors). Next, imposing equality constrains for factor loadings, the configural model was compared with the metric model (M2). Satorra-Bentler scaled-corrected chi-square difference test (Δχ^2^ = 93.00, Δdf = 12, *p* < 0.05) and ΔCFI (≥−0.01) indicated that imposing the equality constraints on factor loading significantly reduced the model fit from the configural model. This could indicate that one or more factors might have differential levels of association with their items for men and women.

**TABLE 5:** Tests of measurement invariance of SASCAT-I across genders for four-factor solutions.

	**Goodness-of-fit indices four-factor solution**					**Difference test indices**						
		
**Model**	**χ2 (*df*)**	**CFI**	**TLI**	**RMSEA (90% CI)**	**SRMR**	**Model comparison**	**Δχ2 (Δ*df*)**	***P* Values**	**ΔCFI**	**ΔRMSEA**	**ΔSRMR**	**Decision**
M1: Configural invariance	661.78^∗∗^ (96)	0.951	0.933	0.031 (0.029–0.033)	0.046	–	–	–	–	–	–	–
M2: Metric invariance	747.965^∗∗^ (108)	0.945	0.932	0.031 (0.029–0.033)	0.048	M1	93.002^∗∗^ (12)	0.000	0.006	0.000	−0.002	Reject
M2a: Partial metric invariance	659.103^∗∗^ (103)	0.952	0.938	0.029 (0.027–0.032)	0.047	M1	10.787 (7)	0.148	−0.001	0.002	−0.001	Accept
M3: Scalar invariance	1599.574^∗∗^ (111)	0.871	0.847	0.046 (0.044–0.048)	0.049	M2a	863.086^∗∗^ (8)	0.000	0.081	−0.017	−0.002	Reject
M3a: Partial scalar invariance	660.237^∗∗^ (104)	0.95	0.939	0.029 (0.027–0.031)	0.047	M2a	1.493 (1)	0.222	0.000	0.000	0.000	Accept

**N* = 12,473; Group 1 (Men) = 5,287; and Group 2 (Women) = 7,186. ^∗^*p* ≤ 0.05, ^∗∗^*p* ≤ 0.01. χ^2^ = chi square statistic; df = degree of freedom; RMSEA = root-mean-square error of approximation; CFI = comparative fit index; TLI = Tucker Lewis index; SRMR = standardized root-mean-square residual.*

As metric invariance was not supported, we examined the modification indices and observed that factor loadings of the cognitive social capital items presented high indices (≥10). We consecutively removed the equality constraint for factor loadings based on the highest modification index and reestimated the metric invariance model. After removing the equality constraint for five items (trust in leaders, trust in stranger, trust in neighbors, sense of belonging, and sense of fairness), the partial metric model (M2a) fitted the data significantly better than the full metric invariance model (Δχ^2^ = 10.79, Δdf = 6, *p* = 0.15; ΔCFI ≤ −0.01; ΔRMSEA ≤ 0.015 and ΔSRMR ≤ 0.03). This indicated that the source of invariance for the factor loading was mostly emerging from the cognitive component of the social capital.

In the next step, scalar invariance (M3) was tested considering intercepts of the items to be equal across genders. However, it resulted in a significantly worse model fit compared to the partial metric model. To achieve a partial scalar invariance, we explored the modification indices and consecutively removed the equality constraints of the intercepts. It required to remove the constraints for 11 items (all except group membership) to achieve a significantly not-worse model fit of the partial scalar invariance (Δχ^2^ = 1.49, Δdf = 1, *p* = 0.222; ΔCFI ≤ −0.01; ΔRMSEA ≤ 0.015 and ΔSRMR ≤ 0.01). This meant practically that the intercept of all items was different across genders. Thus, the MI analysis was not moved forward as the factor structure of social capital was not equivalent for men and women.

### Reestimation of the Factor Structure of Social Capital for Men and Women

After the MI analysis, we were unable to achieve scalar invariance and concluded that the factor structure of social capital measured by the SASCAT-I was not quantitatively similar across genders. To reevaluate the social capital factor structure for each gender–in the final step–a four-factor EFA model was fitted separately among the men (*n* = 2,588) and women (*n* = 3,619) of the first random sample subset. Consecutively, to assess the generalizability of the possible factor structure derived from the EFA models, separate CFA models were fitted with the sample of random subset 2 (men = 2,699 and women = 3,567).

**FIGURE 3:**
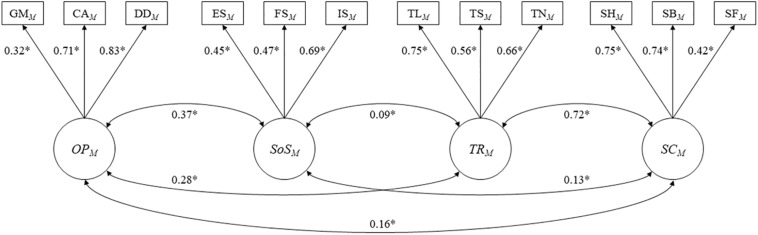
Path diagrams presenting with standradized factor loadings and inter-factor correlations of four-factor CFA model for men (*n* = 2,699). ^∗^*P* < 0.05. Goodness of fit Indices: RMSEA = 0.041, CFI = 0.929, TLI = 0.902. SRMR = 0.058, χ^2^ value = 261.241, df = 48, *p* < 0.01. OP = organizational participation; SoS = social support; TR = trust; SC = social cohesion; GM = group membership; CA = collective action; DD = development discussion, ES = emotional support; FS = financial support; IS = informational support; TS = trust in leaders; TS = trust in strangers; TS = trust in neighbors; SH = social harmony; SB = sense of belonging; SF = sense of fairness; EFA = exploratory factor analysis; CFA = confirmatory factor analysis; RMSEA = root mean square error of approximation; CFI = comparative fit index; TLI = Tucker-Lewis index; SRMR = standardized root mean square residual.

The EFA model for men (*n* = 2,588) identified the same four-factor structure (*organizational participation*, *social support*, *trust*, and *social cohesion*) with good model fit to the data (RMSEA = 0.02, CFI = 0.99, TLI = 0.971, SRMR = 0.032, χ^2^ value = 49.80, df = 24, *p* = 0.0015). The four-factor CFA model for men (*n* = 2,699) also presented a good fit to the data (RMSEA = 0.041, CFI = 0.929, TLI = 0.902, SRMR = 0.058, χ^2^ value = 261.241, df = 48, *p* < 0.01), indicating the generalizability of the EFA model. Figure 3 presents the path diagram showing the standardized factor loadings and interfactor correlations of that model. The standardized factor loadings for individual man’s social capital ranged from 0.32 (group membership) to 0.83 (development discussion), and all factor loadings were significantly different from zero (*p* < 0.05). The interfactor correlation between *organizational participation* and *social support* was 0.37 (*p* < 0.05). On the contrary, cognitive social capital factors (*Trust* and *Social Cohesion*) were highly correlated (correlation coefficient = 0.72, *p* < 0.05).

**FIGURE 4:**
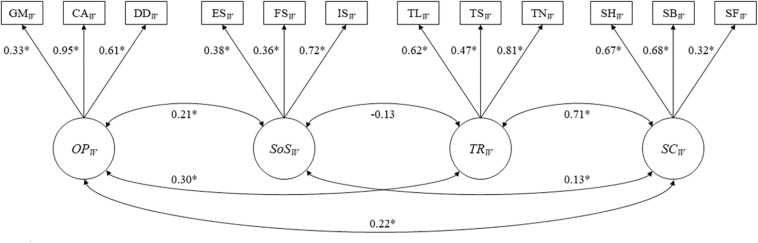
Path diagrams presenting with standradized factor loadings and inter-factor correlations of three-factor CFA model for women (*n* = 3,567). *^∗^P* < 0.05. Goodness of fit Indices: RMSEA = 0.021, CFI = 0.975, TLI = 0.965. SRMR = 0.048, χ^2^ value = 121.974, df = 48, *p* < 0.01. OP = organizational participation; SoS = social support; TR = trust; SC = social cohesion; GM = group membership; CA = collective action; DD = development discussion, ES = emotional support; FS = financial support; IS = informational support; TS = trust in leaders; TS = trust in strangers; TS = trust in neighbors; SH = social harmony; SB = sense of belonging; SF = sense of fairness; EFA = exploratory factor analysis; CFA = confirmatory factor analysis; RMSEA = root mean square error of approximation; CFI = comparative fit index; TLI = Tucker-Lewis index; SRMR = standardized root mean square residual.

The EFA model implemented among the women sample of the random subset one (*n* = 3,619) also identified *Organizational Participation*, *Social Support*, *Trust*, and *Social Cohesion* as the possible factor structure with the necessary model fit indices (RMSEA = 0.011, CFI = 0.996, TLI = 0.99, SRMR = 0.025, χ^2^ value = 35.374, df = 24, *p* < 0.01). Consecutively, the CFA model implemented among the women of the second random subset (*n* = 3,567) also indicated satisfactory goodness of fit (RMSEA = 0.021, CFI = 0.975, TLI = 0.965, SRMR = 0.048, χ^2^ value = 121.974, df = 48, *p* < 0.01), confirming the generalizability of the latent four-factor structure. The standardized factor loadings ranged from 0.32 (sense of fairness) to 0.95 (collective action), and all factor loadings were significantly different from zero (*p* < 0.05). We also observed a high correlation between *Trust* and *Social Cohesion* (correlation coefficient = 0.71, *p* < 0.05). Figure 4 presents the path diagram with the standardized factor loadings and interfactor correlations for the four-factor CFA model for women in the second random subset (*n* = 3,567).

## Discussion

Using a sample population from rural UP, our study explored factor structure and MI of social capital across genders by a short and simple quantitative tool, SASCAT-I. The study concluded, though the social capital of both men and women emerged through four uniquely identified constructs, the structure of the social capital was not the same across genders in the sample population. The result indicated that the factor loadings of *Organizational Participation* and *Social Support* were statistically similar across genders, whereas the loadings of *Trust* and *Social Cohesion* were different. Furthermore, the analysis indicated a partial scalar invariance, implying that item intercepts were not equal across genders.

There are several implications of measurement non-invariance of social capital. First, to conclude that the social capital factors for men and women were equivalent, it was necessary to achieve scalar invariance. Without achieving scalar invariance, comparing latent factor means across genders is not valid. Any factor scores generated while ignoring this issue will also be biased. Second, a partial metric invariance means that men and women perceived the different meaning of the survey questions. Specifically, women and men perceived the construct of *Trust* and *Social Cohesion* differently.

Here, Cook’s (2005) work on trust and social exchange theory can help us unpack how the perception of the cognitive component of social capital may vary across genders. Our results indicated that men in rural UP were much more engaged in economic activity, and they also reported engaging in financial exchange more frequently *via* receiving social support. This financial exchange comes with some uncertainty and obligation (Cook, 2005), where trust (or trustworthiness) matters to evaluate the element of risk that comes with the financial exchange.

According to the social role theory, social interactions, trust, and cohesiveness among men depend on their strategic or agentic behavior, which often focuses on task completion or exchange of resources (Bakan, 1966; Feingold, 1994; Buchan et al., 2008). Therefore, it is essential to distinguish between the different forms of cognitive social capital for men due to the possible independent effects of *Trust* and *Social Cohesion* on financial exchange. Other types of social exchange (such as emotional support or informational support)–in contrast to economic exchange–depends on personal social relations, which are often influenced by the “*act of exchange, not the mode of exchange*” (Cook and Emerson, 1978). In our sample, a significantly higher number of women reported receiving emotional support and informational support. Unlike financial supports, these types of social exchange require cooperative behavior and a higher perception of cohesiveness and harmony (Cross and Markus, 1993), where the distinction between *Trust* and *Social Cohesion* may not be as important. While we are framing our explanation of how men and women may perceive *Trust* and *Social Cohesion*, one critique of our explanations can be that the social exchange theory and social role theory were developed in the context of western culture. However, our arguments still hold in the highly gender inegalitarian patriarchal society of UP (Rajadhyaksha and Velgach, 2015). Though women are getting empowered in India due to education, modernization, and industrialization, gender inequality still prevails in rural UP (Srivastava, 2010).

There are also potential explanations for achieving metric invariance for *Organizational Participation* and *Social Support* across genders. First, in the descriptive analysis, we have found very few respondents who reported to be a part of any community group and few who received financial or informational support from the community. While living in a rural and economically deprived community–irrespective of gender–the respondents may have a limited number of social structures to engage with in the community, which is essential for generating structural social capital. The second explanation is related to how structural social capital questions were framed. In the original SASCAT and in our modified tool, the six indicators related to structural social capital were based on Bourdieu’s network-based resource concept of social capital (Bourdieu, 1986). While exploring the effect of material deprivation and poverty, Lynch et al. (2000) explained that the emergence of structural social capital is much more dependent on socioeconomic inequalities rather than personal characteristics. It is possible, in the context of rural UP, that the dimensions of structural social capital are driven by socioeconomic factors rather than gender. Last, Hox et al. (2015) reported that any survey combining different modes of data collection (such as face-to-face interviews, telephone surveys) might result in non-invariance. Although this study did not implement a mixed-mode survey, the SASCAT-I module was not placed in the same sequence in the questionnaire, which might influence how men and women attach different meanings to the SASCAT-I questions.

Due to the measurement non-invariance, we ended up assessing the factor structure of men and women separately. The result from the gender-stratified analysis demonstrated that, independently, a four-factor solution (*Organizational Participation, Social Support, Trust*, and *Social Cohesion*) of social capital structure was best-fitted for men and women. The stratified models had appropriate goodness of fit with high factor loadings. This means that the measure of social capital derived from SASCAT-I was still valid with appropriate psychometric properties.

In the literature, the theoretical interpretation of social capital through the gender lens indicated that social relationships are not genderless (Leeves and Herbert, 2014; Addis and Joxhe, 2017). Social interaction of an individual with others and others’ behavior toward him or her builds one’s social network. Moreover, because of the inherent difference in the culture and social norms regarding gender, the structure and components of social relationships and networks evolve. Previous studies exploring social capital seldom included a gender dimension. This study included a gender-specific data collection and analytical approach to identify the difference in social capital structure and filled the gap between empirical investigation and theoretical interpretation of the multidimensional and gendered concept of social capital.

### Strengths and Limitations

While multiple cognitive and psychometric validation studies on SASCAT were already being conducted–to the best of our knowledge–our study is the first to examine MI of social capital’s factor structure using psychometric analysis across genders. A large population-based sample of over 12,000 rural respondents was the primary strength of this study. Performing cognitive validation techniques to contextually adapt the scale strengthened our study further. Psychometric techniques such as factor analysis is a model-based approach. As George Box mentioned, “*The most that can be expected from any model is that it can supply a useful approximation to reality: All models are wrong; some models are useful*” (Box et al., 2005). Multiple models with different factor structures can have a set of similar goodness of fit statistics. Hence, the statistical analysis of this study was driven by a robust theoretical framework developed by social capital researchers.

Using a multiple-group CFA approach, we provided further insights into the factor structure of social capital for this population. Contributing to the tradition of establishing MI of any psychosocial constructs, our study addressed the possible measurement bias across genders and advanced the literature of social capital (Stone, 2001; Kawachi et al., 2013; Agampodi et al., 2015; Villalonga-Olives and Kawachi, 2017). On a practical level, the SASCAT-I can measure social capital through a short module within any broader study in a rural population of India. However, calculating a total scale score using this tool by summing the items or taking a mean will produce a biased result. It is necessary to acknowledge the multidimensionality, and the difference in social capital structure across genders and a factor analytical model should be considered for analyzing the data collected by the SASCAT-I.

The result of this study should be interpreted along with its limitations. The sample of our study may be representative of economically disadvantaged rural adults of UP, India; however, the findings of our study may have limited external validity for other settings or among any subset of our sample. Each community and society is unique–thus the process of emergence of social capital in each context. We approached MI having a gender-binary perspective. We decided that this was the best approach to define gender in the context of rural UP. During the analysis, we include the entire sample of the respondents and did not restrict the sample based on any specific criteria (such as age, occupation, or marital status) because we wanted to explore the gender dimension among the entire sample. During data collection, the interviewers were not gender-matched with the respondents, which might affect the way that men and women responded differently to these questions of SASCAT-I. We recommend further cognitive testing, especially for the cognitive social capital component, to explore how data collection processes affect the performance of SASCAT-I. For some of the structural social capital questions (group membership, emotional support, etc.), very few respondents reported positively (yes). While living in a rural and economically deprived community–irrespective of gender–the respondents may have a limited number of social structures to engage with in the community, which is essential for generating structural social capital. Although the lower positive response might lead to a floor effect, the result presented statistically significant factor loadings for all of the structural social capital indicators, demonstrating that they have a well-discriminative capacity to measure social capital.

We also recommend future psychometric exploration of social capital among other social stratifiers. This study only demonstrates MI of social capital measured by SASCAT-I across genders. The survey data collection procedure selected men and women of the households as independent samples. Thus, the assessment of MI between men and women was most logical. Nevertheless, future studies can provide further understandings into the structure of social capital by exploring MI analysis across sociocultural and economic characteristics such as religion, caste, and the socioeconomic class.

Within the scope of the study, we did not explore the reliability of the SASCAT-I. Recent Monte Carlo simulation studies recommended advanced statistical methods to assess reliability for CFA (Geldhof et al., 2014). Applicability of this procedure is limited as they only assess the reliability of unidimensional construct using continuous data. Similarly, the use of other reliability estimates (such as Cronbach’s alpha or Ordinal alpha) will not be appropriate to measure the internal consistency of the 13-item scale as SASCAT-I is multidimensional and contains categorical items.

## Conclusion

Everyday social capital affects the health and well-being of individuals and communities, and acknowledging gender differences in social capital can help us promote equality for women. The body of social capital literature using self-reported measures has limited examples of MI. Addressing this gap, this paper assessed the factor structure and MI across genders for SASCAT-I. Our findings suggest–while the structural components of the social capital (*Organizational Participation* and *Social Support*) have a similar relationship with the corresponding items–the difference in the cognitive component makes social capital of men and women unique. By applying the factor analytical framework, this study provides sufficient evidence regarding the psychometric properties of the SASCAT-I for the rural population of UP, India. However, it is required to perform a gender-stratified analysis to explore the relationship between social capital and other covariates. Moreover, further research is needed to explore the MI of social capital across other sociodemographic factors.

## Data Availability Statement

The raw data supporting the conclusions of this article will be made available by the authors on request to any qualified researcher.

## Ethics Statement

The study involving human participants was reviewed and approved by the Institutional Review Board Office of Johns Hopkins Bloomberg School of Public Health, Baltimore, MD, United States and the Center for Media Studies, New Delhi, India. Written informed consent for participation was not required for this study in accordance with the national legislation and the institutional requirements.

## Author Contributions

MH, J-ML, KR, and SG contributed to the conception and design of the study. MH supervised the data collection, organized the database, and performed the statistical analysis. J-ML, WS, LD, KR, and SG contributed to the interpretation of the results. MH took the lead in writing the manuscript and developed the first draft of the manuscript. All authors contributed to the manuscript revision, and read and approved the submitted version of the manuscript.

## Conflict of Interest

The authors declare that the research was conducted in the absence of any commercial or financial relationships that could be construed as a potential conflict of interest.
