# Supplementary material for: Exploration of Factor Structure and Measurement Invariance by Gender for a Modified Shortened Adapted Social Capital Assessment Tool in India
Source: Front Psychol. 2019 Dec 11;10:2641. doi: 10.3389/fpsyg.2019.02641 (PMC6918543; doi:10.3389/fpsyg.2019.02641)
Supplement: Supplementary file 1 [file Table_1.DOCX]

Supplementary Material

# **1 Rapid Cognitive interview (RCI):**

To improve the validity of the English SASCAT questions, the study implemented a modified version of the cognitive interview. Cognitive interviewing^[[1]](#footnote-1)^ is “the administration of draft survey questions while collecting additional verbal information about the survey responses, which is used to evaluate the quality of the response or to help determine whether the question is generating the information that its author intends.” This is a process of asking the participant of the interview to provide additional information about their perception of the question. While performing a full conative interview much more elaborative, the qualitative data collection process is implemented, and the cognitive interview continues until the near situation of the data is achieved (i.e., the respondents understand the questions quickly, consistently, and correctly). However, in the context of the broader study and this dissertation research implementing a full-fledged cognitive validation study was not possible due to the resource limitation.

Between the two ways of performing conative interview (thinking aloud vs. verbal probing) we have adopted probing to conduct a shortened version, “rapid cognitive interviewing,” to elucidate the understanding of questions by the participant and further improve the wording, phrasing and the categories of SASCAT. The RCI was implemented among the household heads in the local community structured as an informal discussion by moderators experienced in qualitative data collection. A team consisting one moderator and note taker initially approached potential participants to the RCI. They received verbal informed consent, and no personally identifiable data were collected from the participants. After receiving consent, the following steps were performed:

1. Introduction: The moderator explains the objective of the discussion
2. Question: Fist the initial translated question was asked to the participant
3. Individual comprehension/interpretation: The moderator asked explanations of some specific words from the question
4. Paraphrasing: The moderator asked, “If you were asking this question to your neighbor, how would you say it?”
5. Contextual comprehension/interpretation: The moderator as “Would this question be easy or hard for your neighbor to answer?” (If hard) “Why would it be hard to answer?”
6. The moderator repeats the step b, c, d and e for the next question.

Both moderator and the note taker write down extensive field notes and debrief the study team about the findings. According to the finding of the RCI further modification of the questions are performed. Below one illustrative RCI question is presented.

**Rapid cognitive interviewing (RCI) verbal probing steps:**

- Ask the respondent:

*Original question: Do you feel that this village/area is yours?*

- - *Yes*
  - *Sometime*
  - *No*
- Individual comprehension/interpretation:
  - *“Can you explain what these words mean to you: What is the meaning of “feeling something is yours”? Is “village” and “area” means the same thing?”*
- Paraphrasing:
  - *“If you were asking this question to your neighbor, how would you say it?”*
- Contextual comprehension/interpretation:
  - *“Would this question be easy or hard for your neighbor to answer?” (If hard) “Why would it be hard to answer?”*
- After performing the RCI and performing adequate changes in the question we finalized this question as:
  - *“Do you feel that you have a sense of belonging to this village?”*

# **2 SASCAT, SASCAT-B and modified SASCAT-India (SASCAT-I)**

|  | **SASCAT** | **SASCAT-B** | | **SASCAT-I** | |
| --- | --- | --- | --- | --- | --- |
|  | ***Group Membership*** | | | | |
| **Structural Social Capital** | 1. In the last 12 months have you been an active member of any of the following types of groups in your community?   - Work related/trade union - Community association/co-op - Women’s group - Political group - Religious group - Credit/funeral group - Sports group - Other: specify | 1a. In the last 12 months, have you been a member of the following types of groups in your area?   - Vocational training group - Savings group/community cooperative - Political group - Religious group - Microcredit program - Sports club - Youth/student club - Other: specify | | 1a. In the last 12 months have you been a member of any of the following groups?   - Group from where you can get loans (Microcredit group) - Group from which you can receive any training - Self-help Groups (SHG) - Any other women group - Farmer/ Fisherman/ Cattle herder’s group - Businessman/ Professionals group - Religious group - Gram panchayat and its committee - Village Council - Village health, sanitation, and nutrition committee - School management committee - Youth/student group or club - Political group - Other: specify | |
|  |  | 1b. In the last 12 months, how would you describe your involvement in the groups in which you are a member?   - Received a loan or other form of financial support - Attended meetings - Attended training - Participated in decision making - Served as a leader of the group - Other: specify | | 1b. In the last 12 months, how have you participated in or benefited from the group?   - Received a loan or other form of financial support - Received any in-kind support (other than financial) - Provide Financial contribution to the group - Attended meetings - Attended training - Participated in decision making - Served as a leader of the group - Other: specify | |
|  | ***Social Support*** | | | | |
|  | 2. In the last 12 months, did you receive from the group any emotional help, economic help or assistance in helping you know or do things?   - Work related/trade union - Community association/co-op - Women’s group - Political group - Religious group - Credit/funeral group - Sports group - Other: specify | 2a. Suppose you had something unfortunate happen to you, such as a father’s sudden death. Who would help you in this situation?   - Immediate family - Relatives - Neighbors - Friends who are not neighbors - Community leaders - Religious leaders - Politicians - Government officials/civil service - Person from NGO - A group in which I am a member - A group in which I am not a member - Other: specify | | 2. In the last 12 months, did you receive any emotional support any sudden or unfortunate event (like a death of a family member or other troubling event) from any of the following people?   - Immediate family - Relatives - Neighbors - Friends (not -neighbor) - Co-workers - Healthcare provider - Leaders of the Village - Religious leaders - Political Leaders - Government officials - Personnel from NGO - Other: specify | |
|  | **SASCAT** | | **SASCAT-B** | | **SASCAT-I** |
| **Structural Social Capital** | 3. In the last 12 months, have you received any help or support from any of the following, this can be emotional help, economic help or assistance in helping you know or do things?   - Family - Neighbors - Friends who are not neighbors - Community leaders - Religious leaders - Politicians - Government officials/civil service - Charitable organizations/NGO - Other: specify | | 2b. Suppose you suffered an economic loss, such as job loss (urban)/crop failure (rural). In that situation, who do you think would assist you financially^a^? | | 3. In the last 12 months, did you receive any financial support for any economic loss (such as crop failure, loss of livestock, loss of a job, or something similar) loss from any of the following people? This may include receiving or borrowing money^b^. |
|  |  |  | 2c. Suppose you are (female)/your wife is (male) preparing to give birth to your (female)/her (male) first child. Who do you think would provide you (female)/her (male) advice or assistance in this situation^a^? | | 4. In the last 12 months, did you receive any support, advice or assistance from any of the following people for seeking healthcare (Such as taking a family member or friend to the doctor or hospital)^b^ ? |
|  | ***Collective Action*** | | | | |
|  | 4. In the last 12 months, have you joined together with other community members to address a problem or common issue?   - Yes - No | | 3. In the last 12 months, have you joined together with others in your area to address important issues?   - Yes - No | | 5. In the last 12 months, have you worked together with other community members and attempted to address a problem or common issue of the village?   - Yes - No |
|  | 5. In the last 12 months, have you talked to a local authority or governmental organization about problems in this community?   - Yes - No | | 4. In the last 12 months, have you talked with a local leader, chairman, or governmental organization about the development of your area?   - Yes - No | | 6. In the past 12 months, have you spoken with anyone about the development of your village?   - Leaders of the Village - Religious leaders - Political Leaders - Government officials - Personnel from NGO - Others: specify |
| **Cognitive Social Capital** | ***Trust*** | |  | |  |
|  | 6. In general, can the majority of people in this community be trusted?   - Yes - No | | 5a. Can your neighbors be trusted?   - Yes - Sometimes - No | | 7. Overall, do you think the leaders of your village can be trusted?   - Yes - Sometimes - No |
|  |  |  | 5b. Can leaders in this area be trusted?   - Yes - Sometimes - No | | 8. Overall, do you think your neighbors in your village can be trusted?   - Yes - Sometimes - No |
|  |  |  |  | | 9. Overall, do you think people you are not familiar residing in your village can be trusted?   - Yes - Sometimes - No |
|  | **SASCAT** | | **SASCAT-B** | | **SASCAT-I** |
|  | ***Social Cohesion*** | |  | |  |
| **Cognitive Social Capital** | 7. Do you think that the majority of people in this community would try to take advantage of you if they got the chance?   - Yes - No | | 6. Do you think that the majority of people in this area would try to take advantage of you if they got the chance?   - Yes - Sometimes - No | | 10. Do you think that the majority of people in your village would try to take advantage of you if they got the chance?   - Yes - Sometimes - No |
|  | 8. Do the majority of people in this community generally get along with each other?   - Yes - No | | 7. Do the majority of people in this area generally have good relationships with each other?   - Yes - Sometimes - No | | 11. Do you think the majority of people in this village generally have good relationships with each other?   - Yes - Sometimes - No |
|  | 9. Do you feel as though you are really a part of this community?   - Yes - No | | 8. Do you feel that this area is yours?   - Yes - Sometimes - No | | 12. Do you feel that you have a sense of belonging to this village?   - Yes - Sometimes - No |
| Note: ^a^Use the same list of response options as in Question 2a.  ^b^Use the same list of response options as in Question 2. | | | | | |

# **3 Polychoric correlation of 12 social capital items generated from SASCAT-I**

| Items | | Organizational Participation | | | Social Support | | | Trust | | | Social  Cohesion | | |
| --- | --- | --- | --- | --- | --- | --- | --- | --- | --- | --- | --- | --- | --- |
|  |  | GM | CA | DD | ES | FS | IS | TL | TN | TS | SCR | SCSB | SCF |
|  | | Polychoric correlation of the total sample (n= 12,473) | | | | | | | | | | | |
| Structural Component | GM | **1** |  |  |  |  |  |  |  |  |  |  |  |
|  | CA | 0.22 | **1** |  |  |  |  |  |  |  |  |  |  |
|  | DD | 0.23 | 0.62 | **1** |  |  |  |  |  |  |  |  |  |
|  | ES | 0.08 | 0.11 | 0.13 | **1** |  |  |  |  |  |  |  |  |
|  | FS | 0.07 | 0.10 | 0.16 | 0.21 | **1** |  |  |  |  |  |  |  |
|  | IS | 0.09 | 0.07 | 0.12 | 0.26 | 0.21 | **1** |  |  |  |  |  |  |
| Cognitive Component | TL | 0.08 | 0.21 | 0.16 | 0.03 | 0.00 | -0.02 | **1** |  |  |  |  |  |
|  | TN | 0.05 | 0.09 | 0.09 | -0.01 | 0.00 | -0.01 | 0.49 | **1** |  |  |  |  |
|  | TS | 0.05 | 0.27 | 0.16 | 0.00 | 0.01 | -0.06 | 0.40 | 0.33 | **1** |  |  |  |
|  | SH | 0.08 | 0.16 | 0.09 | -0.01 | 0.01 | -0.01 | 0.33 | 0.45 | 0.20 | **1** |  |  |
|  | SB | 0.04 | 0.02 | 0.07 | -0.04 | 0.12 | 0.12 | 0.27 | 0.44 | 0.16 | 0.51 | **1** |  |
|  | SF | 0.05 | 0.16 | 0.07 | 0.06 | 0.04 | 0.11 | 0.14 | 0.18 | 0.14 | 0.24 | 0.29 | **1** |
|  | | Polychoric correlation of the sample of women in upper triangle (n= 7,186) | | | | | | | | | | | |
| Structural Component | GM | **1** | 0.21 | 0.20 | 0.08 | 0.06 | 0.09 | 0.12 | 0.07 | 0.05 | 0.10 | 0.08 | 0.05 |
|  | CA | 0.26 | **1** | 0.56 | 0.08 | 0.15 | 0.06 | 0.16 | 0.13 | 0.23 | 0.18 | 0.06 | 0.08 |
|  | DD | 0.30 | 0.61 | **1** | 0.16 | 0.09 | 0.13 | 0.15 | 0.08 | 0.14 | 0.05 | 0.03 | 0.01 |
|  | ES | 0.08 | 0.16 | 0.13 | **1** | 0.18 | 0.24 | 0.04 | 0.01 | 0.02 | 0.01 | -0.03 | 0.03 |
|  | FS | 0.08 | 0.06 | 0.20 | 0.27 | **1** | 0.18 | 0.03 | -0.05 | 0.02 | 0.04 | 0.08 | 0.00 |
|  | IS | 0.09 | 0.14 | 0.18 | 0.29 | 0.26 | **1** | -0.04 | -0.03 | -0.10 | 0.02 | 0.15 | 0.09 |
| Cognitive Component | TL | 0.03 | 0.20 | 0.13 | 0.04 | -0.04 | 0.02 | **1** | 0.50 | 0.31 | 0.29 | 0.27 | 0.10 |
|  | TN | 0.03 | 0.02 | 0.04 | -0.03 | 0.06 | 0.06 | 0.47 | **1** | 0.35 | 0.44 | 0.42 | 0.13 |
|  | TS | 0.06 | 0.26 | 0.12 | -0.02 | 0.00 | 0.03 | 0.48 | 0.27 | **1** | 0.20 | 0.14 | 0.07 |
|  | SH | 0.06 | 0.10 | 0.07 | -0.03 | -0.03 | -0.03 | 0.36 | 0.45 | 0.16 | **1** | 0.46 | 0.23 |
|  | SB | -0.01 | -0.07 | 0.04 | -0.03 | 0.18 | 0.11 | 0.23 | 0.44 | 0.15 | 0.56 | **1** | 0.24 |
|  | SF | 0.05 | 0.16 | 0.09 | 0.15 | 0.08 | 0.18 | 0.19 | 0.23 | 0.20 | 0.23 | 0.37 | **1** |
|  | | Polychoric correlation of the sample of men in lower triangle (n= 5,287) | | | | | | | | | | | |
| Note: GM = Group Membership, CA = Collective Action, DD = Development Discussion, ES = Emotional Support,  FS = Financial Support, IS = Informational Support, TS = Trust in Leaders, TS = Trust in Strangers, TS = Trust in Neighbors,  SH = Social Harmony, SB = Sense of Belonging, SF = Sense of Fairness   \|  \| Correlation between items from the same constructs \| \| --- \| --- \| \|  \| Correlation between items from the same social capital constructs but within same components \| \|  \| of social capital (Structural and Cognitive) \| \|  \| Correlation between items from the different social capital constructs with different social capital \| \|  \| component \| | | | | | | | | | | | | | |

# **4 MPLUS code for Measurement Invariance Analysis:**

## **Exploratory Factor Analysis Disregarding Gender: Four Factor Solution**

TITLE:

Single level EFA with four correlated continuous factors WLSMV estimate for first

randomly selected group (rand group = 1)

DATA:

File = "~\SASCAT4.txt";

VARIABLE:

Names are

adult adultm adultw rand randsex arandsex memid hhid district block

Position Sex Age Education Occupation Marriage HHreligion HHcaste

HHwealthindex GMB GMBB CA DD DDB SSE SSEB SSF SSFB SSI SSIB TRUSTL

TRUSTN TRUSTS SCH SCB SCF householdmemberid respuniqueid psu;

Missing are all (-9999) ;

USEVARIABLES = GMBB CA DDB SSEB SSFB SSIB

TRUSTL TRUSTN TRUSTS SCH SCB SCF;

CATEGORICAL = GMBB CA DDB SSEB SSFB SSIB

TRUSTL TRUSTN TRUSTS SCH SCB SCF;

USEOBSERVATION ARE (rand EQ 1);

ANALYSIS:

TYPE = EFA 4 4;

OUTPUT:

MODINDICES;

TECH1;

standardized;

## **Confirmatory Factor Analysis Disregarding Gender: Four Factor Solution**

TITLE:

Single level CFA with four continuous correlated factors WLSMV estimate for second

randomly selected group (rand group = 2)

DATA:

File = "~\SASCAT4.txt";

VARIABLE:

Names are

adult adultm adultw rand randsex arandsex memid hhid district block

Position Sex Age Education Occupation Marriage HHreligion HHcaste

HHwealthindex GMB GMBB CA DD DDB SSE SSEB SSF SSFB SSI SSIB TRUSTL

TRUSTN TRUSTS SCH SCB SCF householdmemberid respuniqueid psu;

Missing are all (-9999) ;

USEVARIABLES = GMBB CA DDB SSEB SSFB SSIB

TRUSTL TRUSTN TRUSTS SCH SCB SCF;

CATEGORICAL = GMBB CA DDB SSEB SSFB SSIB

TRUSTL TRUSTN TRUSTS SCH SCB SCF;

USEOBSERVATION ARE (rand EQ 2);

Analysis:

Estimator = WLSMV;

MODEL:

f1 BY GMBB* CA DDB;

f2 BY SSEB* SSFB SSIB;

f3 BY TRUSTL* TRUSTN TRUSTS;

f4 BY SCH* SCB SCF;

f1@1;

f2@1;

f3@1;

f4@1;

OUTPUT:

TECH1;

standardized;

SAVEDATA:

DIFFTEST IS aps_4FWLSMV.dat;

## **Measurement Invariance Test Step 1: Configural Model**

TITLE:

Measurement Invariance Test Step 1: Configural Model

Single level CFA with Four correlated continious factors WLSMV estimate

DATA:

File = "~\SASCAT4.txt";

Variable:

Names are

adult adultm adultw rand randsex arandsex memid hhid district block

Position Sex Age Education Occupation Marriage HHreligion HHcaste

HHwealthindex GMB GMBB CA DD DDB SSE SSEB SSF SSFB SSI SSIB TRUSTL

TRUSTN TRUSTS SCH SCB SCF householdmemberid respuniqueid psu;

Missing are all (-9999) ;

USEVARIABLES = GMBB CA DDB SSEB SSFB SSIB

TRUSTL TRUSTN TRUSTS SCH SCB SCF;

CATEGORICAL = GMBB CA DDB SSEB SSFB SSIB

TRUSTL TRUSTN TRUSTS SCH SCB SCF;

GROUPING IS Sex (1=Men 2=Women);

ANALYSIS:

ESTIMATOR = WLSMV;

COVERAGE = .00005;

ITERATIONS = 100000;

PARAMETERIZATION=THETA;

OUTPUT: SAMPSTAT;

MODINDICES;

STDYX;

MODEL:

!MODEL1: Reference Group (Men)

!Factor Loadings ALL FREE estimated

f1 BY GMBB* (1)

CA* (2)

DDB* (3);

f2 BY SSEB* (4)

SSFB* (5)

SSIB* (6);

f3 BY TRUSTL* (7)

TRUSTN* (8)

TRUSTS* (9);

f4 BY SCH* (10)

SCB* (11)

SCF* (12);

!Item thresholds all free

[GMBB$1];

[CA$1];

[DDB$1];

[SSEB$1];

[SSFB$1];

[SSIB$1];

[TRUSTL$1];

[TRUSTL$2];

[TRUSTN$1];

[TRUSTN$2];

[TRUSTS$1];

[TRUSTS$2];

[SCH$1];

[SCH$2];

[SCB$1];

[SCB$2];

[SCF$1];

[SCF$2];

!Factor means (fixed=0 for identification)

[f1@0];

[f2@0];

[f3@0];

[f4@0];

!Factor variances (fix=1 for identification)

f1@1;

f2@1;

f3@1;

f4@1;

!Item Residual Variances all fixed = 1

GMBB@1 CA@1 DDB@1 SSEB@1 SSFB@1 SSIB@1

TRUSTL@1 TRUSTN@1 TRUSTS@1 SCH@1 SCB@1 SCF@1;

!Model 2: CONFIGUAL MODEL FOR FEMALE GROUP

MODEL Women:

!Factor Loadings-FREE ESTIMATED

f1 BY GMBB*

CA*

DDB*;

f2 BY SSEB*

SSFB*

SSIB*;

f3 BY TRUSTL*

TRUSTN*

TRUSTS*;

f4 BY SCH*

SCB*

SCF*;

!Thresholds all free.

[GMBB$1];

[CA$1];

[DDB$1];

[SSEB$1];

[SSFB$1];

[SSIB$1];

[TRUSTL$1];

[TRUSTL$2];

[TRUSTN$1];

[TRUSTN$2];

[TRUSTS$1];

[TRUSTS$2];

[SCH$1];

[SCH$2];

[SCB$1];

[SCB$2];

[SCF$1];

[SCF$2];

!Factor means (fixed=0 for identification)

[f1@0];

[f2@0];

[f3@0];

[f4@0];

!Factor variances (fix=1 for identification);

f1@1;

f2@1;

f3@1;

f4@1;

!Residual Variances all fixed @1

GMBB@1 CA@1 DDB@1 SSEB@1 SSFB@1 SSIB@1

TRUSTL@1 TRUSTN@1 TRUSTS@1 SCH@1 SCB@1 SCF@1;

SAVEDATA: DIFFTEST IS aps_configual.dat;

## **Measurement Invariance Test Step 2: Metric or Weak Factorial Invariance Model**

TITLE:

Measurement Invariance Test Step 2: Metric Invariance Model

Single level CFA with four correlated continious factors WSMV estimate

DATA:

File = "~\SASCAT4.txt";

Variable:

Names are

adult adultm adultw rand randsex arandsex memid hhid district block

Position Sex Age Education Occupation Marriage HHreligion HHcaste

HHwealthindex GMB GMBB CA DD DDB SSE SSEB SSF SSFB SSI SSIB TRUSTL

TRUSTN TRUSTS SCH SCB SCF householdmemberid respuniqueid psu;

Missing are all (-9999) ;

USEVARIABLES = GMBB CA DDB SSEB SSFB SSIB

TRUSTL TRUSTN TRUSTS SCH SCB SCF;

CATEGORICAL = GMBB CA DDB SSEB SSFB SSIB

TRUSTL TRUSTN TRUSTS SCH SCB SCF;

GROUPING IS Sex (1=Men 2=Women);

ANALYSIS:

ESTIMATOR = WLSMV;

COVERAGE = .00005;

ITERATIONS = 100000;

PARAMETERIZATION=THETA;

DIFFTEST IS aps_configual.dat;

OUTPUT: SAMPSTAT;

MODINDICES;

STDYX;

MODEL:

!MODEL1: Reference Group (Men)

!Factor Loadings ALL FREE estimated

f1 BY GMBB* (1)

CA* (2)

DDB* (3);

f2 BY SSEB* (4)

SSFB* (5)

SSIB* (6);

f3 BY TRUSTL* (7)

TRUSTN* (8)

TRUSTS* (9);

f4 BY SCH* (10)

SCB* (11)

SCF* (12);

!Item thresholds all free

[GMBB$1];

[CA$1];

[DDB$1];

[SSEB$1];

[SSFB$1];

[SSIB$1];

[TRUSTL$1];

[TRUSTL$2];

[TRUSTN$1];

[TRUSTN$2];

[TRUSTS$1];

[TRUSTS$2];

[SCH$1];

[SCH$2];

[SCB$1];

[SCB$2];

[SCF$1];

[SCF$2];

!Factor means (fixed=0 for identification)

[f1@0];

[f2@0];

[f3@0];

[f4@0];

!Factor variances (fix=1 for identification)

f1@1;

f2@1;

f3@1;

f4@1;

!Item Residual Variances all fixed = 1

GMBB@1 CA@1 DDB@1 SSEB@1 SSFB@1 SSIB@1

TRUSTL@1 TRUSTN@1 TRUSTS@1 SCH@1 SCB@1 SCF@1;

!Model 2: CONFIGUAL MODEL FOR FEMALE GROUP

MODEL Women:

!Factor Loadings-ALL EQUAL TO MALE

f1 BY GMBB* (1)

CA* (2)

DDB* (3);

f2 BY SSEB* (4)

SSFB* (5)

SSIB* (6);

f3 BY TRUSTL* (7)

TRUSTN* (8)

TRUSTS* (9);

f4 BY SCH* (10)

SCB* (11)

SCF* (12);

!Thresholds all free.

[GMBB$1];

[CA$1];

[DDB$1];

[SSEB$1];

[SSFB$1];

[SSIB$1];

[TRUSTL$1];

[TRUSTL$2];

[TRUSTN$1];

[TRUSTN$2];

[TRUSTS$1];

[TRUSTS$2];

[SCH$1];

[SCH$2];

[SCB$1];

[SCB$2];

[SCF$1];

[SCF$2];

!Factor means (fixed=0 for identification)

[f1@0];

[f2@0];

[f3@0];

[f4@0];

!Factor variances (NOW FREE);

f1@1;

f2@1;

f3@1;

f4@1;

!Residual Variances all fixed @1

GMBB@1 CA@1 DDB@1 SSEB@1 SSFB@1 SSIB@1

TRUSTL@1 TRUSTN@1 TRUSTS@1 SCH@1 SCB@1 SCF@1;

SAVEDATA:

DIFFTEST IS aps_metric.dat;

## **Measurement Invariance Test Step 3: Partial Metric or Partial Weak Factorial Invariance Model**

TITLE:

Measurement Invariance Test Step 3: Partial Weak Factorial Invariance Model

Single level CFA with four correlated continious factors WSMV estimate

DATA:

File = "~\SASCAT4.txt";

Variable:

Names are

adult adultm adultw rand randsex arandsex memid hhid district block

Position Sex Age Education Occupation Marriage HHreligion HHcaste

HHwealthindex GMB GMBB CA DD DDB SSE SSEB SSF SSFB SSI SSIB TRUSTL

TRUSTN TRUSTS SCH SCB SCF householdmemberid respuniqueid psu;

Missing are all (-9999) ;

USEVARIABLES = GMBB CA DDB SSEB SSFB SSIB

TRUSTL TRUSTN TRUSTS SCH SCB SCF;

CATEGORICAL = GMBB CA DDB SSEB SSFB SSIB

TRUSTL TRUSTN TRUSTS SCH SCB SCF;

GROUPING IS Sex (1=Men 2=Women);

ANALYSIS:

ESTIMATOR = WLSMV;

COVERAGE = .00005;

ITERATIONS = 100000;

PARAMETERIZATION=THETA;

DIFFTEST IS aps_configual.dat;

OUTPUT: SAMPSTAT;

MODINDICES;

STDYX;

MODEL:

!MODEL1: Reference Group (Men)

!Factor Loadings ALL FREE estimated

f1 BY GMBB* (1)

CA* (2)

DDB* (3);

f2 BY SSEB* (4)

SSFB* (5)

SSIB* (6);

f3 BY TRUSTL* (7)

TRUSTN* (8)

TRUSTS* (9);

f4 BY SCH* (10)

SCB* (11)

SCF* (12);

!Item thresholds all free

[GMBB$1];

[CA$1];

[DDB$1];

[SSEB$1];

[SSFB$1];

[SSIB$1];

[TRUSTL$1];

[TRUSTL$2];

[TRUSTN$1];

[TRUSTN$2];

[TRUSTS$1];

[TRUSTS$2];

[SCH$1];

[SCH$2];

[SCB$1];

[SCB$2];

[SCF$1];

[SCF$2];

!Factor means (fixed=0 for identification)

[f1@0];

[f2@0];

[f3@0];

[f4@0];

!Factor variances (fix=1 for identification)

f1@1;

f2@1;

f3@1;

f4@1;

!Item Residual Variances all fixed = 1

GMBB@1 CA@1 DDB@1 SSEB@1 SSFB@1 SSIB@1

TRUSTL@1 TRUSTN@1 TRUSTS@1 SCH@1 SCB@1 SCF@1;

!Model 2: CONFIGUAL MODEL FOR FEMALE GROUP

MODEL Women:

!Factor Loadings-ALL EQUAL TO MALE

f1 BY GMBB* (1)

CA* (2)

DDB* (3);

f2 BY SSEB* (4)

SSFB* (5)

SSIB* (6);

f3 BY TRUSTL*

TRUSTN*

TRUSTS* ;

f4 BY SCH* (10)

SCB*

SCF* ;

!Thresholds all free.

[GMBB$1];

[CA$1];

[DDB$1];

[SSEB$1];

[SSFB$1];

[SSIB$1];

[TRUSTL$1];

[TRUSTL$2];

[TRUSTN$1];

[TRUSTN$2];

[TRUSTS$1];

[TRUSTS$2];

[SCH$1];

[SCH$2];

[SCB$1];

[SCB$2];

[SCF$1];

[SCF$2];

!Factor means (fixed=0 for identification)

[f1@0];

[f2@0];

[f3@0];

[f4@0];

!Factor variances (NOW FREE);

f1@1;

f2@1;

f3@1;

f4@1;

!Residual Variances all fixed @1

GMBB@1 CA@1 DDB@1 SSEB@1 SSFB@1 SSIB@1

TRUSTL@1 TRUSTN@1 TRUSTS@1 SCH@1 SCB@1 SCF@1;

SAVEDATA:

DIFFTEST IS aps_partialmetric.dat;

## **Measurement Invariance Test Step 4: Scalar or Strong Factorial Invariance Model**

TITLE:

Measurement Invariance Test Step 3: Scalar Invariance Model

Single level CFA with four correlated continious factors WSMV estimate

DATA:

File = "~\SASCAT4.txt";

Variable:

Names are

adult adultm adultw rand randsex arandsex memid hhid district block

Position Sex Age Education Occupation Marriage HHreligion HHcaste

HHwealthindex GMB GMBB CA DD DDB SSE SSEB SSF SSFB SSI SSIB TRUSTL

TRUSTN TRUSTS SCH SCB SCF householdmemberid respuniqueid psu;

Missing are all (-9999) ;

USEVARIABLES = GMBB CA DDB SSEB SSFB SSIB

TRUSTL TRUSTN TRUSTS SCH SCB SCF;

CATEGORICAL = GMBB CA DDB SSEB SSFB SSIB

TRUSTL TRUSTN TRUSTS SCH SCB SCF;

GROUPING IS Sex (1=Men 2=Women);

ANALYSIS:

ESTIMATOR = WLSMV;

COVERAGE = .00005;

ITERATIONS = 100000;

PARAMETERIZATION=THETA;

DIFFTEST IS aps_partialmetric.dat;

OUTPUT: SAMPSTAT;

MODINDICES;

STDYX;

MODEL:

!MODEL1: Reference Group (Male)

!Factor Loadings ALL FREE estimated

f1 BY GMBB* (1)

CA* (2)

DDB* (3);

f2 BY SSEB* (4)

SSFB* (5)

SSIB* (6);

f3 BY TRUSTL* (7)

TRUSTN* (8)

TRUSTS* (9);

f4 BY SCH* (10)

SCB* (11)

SCF* (12);

!Item thresholds all free

[GMBB$1];

[CA$1];

[DDB$1];

[SSEB$1];

[SSFB$1];

[SSIB$1];

[TRUSTL$1];

[TRUSTL$2];

[TRUSTN$1];

[TRUSTN$2];

[TRUSTS$1];

[TRUSTS$2];

[SCH$1];

[SCH$2];

[SCB$1];

[SCB$2];

[SCF$1];

[SCF$2];

!Factor means (fixed=0 for identification)

[f1@0];

[f2@0];

[f3@0];

[f4@0];

!Factor variances (fix=1 for identification)

f1@1;

f2@1;

f3@1;

f4@1;

!Item Residual Variances all fixed = 1

GMBB@1 CA@1 DDB@1 SSEB@1 SSFB@1 SSIB@1

TRUSTL@1 TRUSTN@1 TRUSTS@1 SCH@1 SCB@1 SCF@1;

!Model 2: CONFIGUAL MODEL FOR FEMALE GROUP

MODEL Women:

!Factor Loadings-ALL EQUAL TO MALE

f1 BY GMBB* (1)

CA* (2)

DDB* (3);

f2 BY SSEB* (4)

SSFB* (5)

SSIB* (6);

f3 BY TRUSTL*

TRUSTN*

TRUSTS* ;

f4 BY SCH* (10)

SCB*

SCF* ;

! Selected Item Thresholds NOW HELD EQUAL IF LEFT BLANK

[TRUSTL$1];

[TRUSTL$2];

[TRUSTN$1];

[TRUSTN$2];

[TRUSTS$1];

[TRUSTS$2];

[SCB$1];

[SCB$2];

[SCF$1];

[SCF$2];

!Factor means (fixed=0 for identification)

[f1@0];

[f2@0];

[f3@0];

[f4@0];

!Factor variances (fix=1 for identification)

f1@1;

f2@1;

f3@1;

f4@1;

!Residual Variances all fixed @1

GMBB@1 CA@1 DDB@1 SSEB@1 SSFB@1 SSIB@1

TRUSTL@1 TRUSTN@1 TRUSTS@1 SCH@1 SCB@1 SCF@1;

SAVEDATA:

DIFFTEST IS aps_scalar.dat;

## **Measurement Invariance Test Step 5: Partial Scalar or Strong Factorial Invariance Model**

TITLE:

Measurement Invariance Test Step 3: partial Scalar Invariance Model

Single level CFA with four correlated continious factors WSMV estimate

DATA:

File = "~\SASCAT4.txt";

Variable:

Names are

adult adultm adultw rand randsex arandsex memid hhid district block

Position Sex Age Education Occupation Marriage HHreligion HHcaste

HHwealthindex GMB GMBB CA DD DDB SSE SSEB SSF SSFB SSI SSIB TRUSTL

TRUSTN TRUSTS SCH SCB SCF householdmemberid respuniqueid psu;

Missing are all (-9999) ;

USEVARIABLES = GMBB CA DDB SSEB SSFB SSIB

TRUSTL TRUSTN TRUSTS SCH SCB SCF;

CATEGORICAL = GMBB CA DDB SSEB SSFB SSIB

TRUSTL TRUSTN TRUSTS SCH SCB SCF;

GROUPING IS Sex (1=Men 2=Women);

ANALYSIS:

ESTIMATOR = WLSMV;

COVERAGE = .00005;

ITERATIONS = 100000;

PARAMETERIZATION=THETA;

DIFFTEST IS aps_partialmetric.dat;

OUTPUT: SAMPSTAT;

MODINDICES;

STDYX;

MODEL:

!MODEL1: Reference Group (Male)

!Factor Loadings ALL FREE estimated

f1 BY GMBB* (1)

CA* (2)

DDB* (3);

f2 BY SSEB* (4)

SSFB* (5)

SSIB* (6);

f3 BY TRUSTL* (7)

TRUSTN* (8)

TRUSTS* (9);

f4 BY SCH* (10)

SCB* (11)

SCF* (12);

!Item thresholds all free

[GMBB$1];

[CA$1];

[DDB$1];

[SSEB$1];

[SSFB$1];

[SSIB$1];

[TRUSTL$1];

[TRUSTL$2];

[TRUSTN$1];

[TRUSTN$2];

[TRUSTS$1];

[TRUSTS$2];

[SCH$1];

[SCH$2];

[SCB$1];

[SCB$2];

[SCF$1];

[SCF$2];

!Factor means (fixed=0 for identification)

[f1@0];

[f2@0];

[f3@0];

[f4@0];

!Factor variances (fix=1 for identification)

f1@1;

f2@1;

f3@1;

f4@1;

!Item Residual Variances all fixed = 1

GMBB@1 CA@1 DDB@1 SSEB@1 SSFB@1 SSIB@1

TRUSTL@1 TRUSTN@1 TRUSTS@1 SCH@1 SCB@1 SCF@1;

!Model 2: CONFIGUAL MODEL FOR FEMALE GROUP

MODEL Women:

!Factor Loadings-ALL EQUAL TO MALE

f1 BY GMBB* (1)

CA* (2)

DDB* (3);

f2 BY SSEB* (4)

SSFB* (5)

SSIB* (6);

f3 BY TRUSTL*

TRUSTN*

TRUSTS* ;

f4 BY SCH* (10)

SCB*

SCF* ;

! Selected Item Thresholds NOW HELD EQUAL IF LEFT BLANK

[TRUSTL$1];

[TRUSTL$2];

[TRUSTN$1];

[TRUSTN$2];

[TRUSTS$1];

[TRUSTS$2];

[SCB$1];

[SCB$2];

[SCF$1];

[SCF$2];

[SCH$1];

[SCH$2];

[SSEB$1];

[CA$1];

[DDB$1];

[SSFB$1];

[SSIB$1];

!Factor means (fixed=0 for identification)

[f1@0];

[f2@0];

[f3@0];

[f4@0];

!Factor variances (fix=1 for identification)

f1@1;

f2@1;

f3@1;

f4@1;

!Residual Variances all fixed @1

GMBB@1 CA@1 DDB@1 SSEB@1 SSFB@1 SSIB@1

TRUSTL@1 TRUSTN@1 TRUSTS@1 SCH@1 SCB@1 SCF@1;

SAVEDATA:

DIFFTEST IS aps_partialscalar.dat;

# **5 MPLUS code for Gender Sratified Factor Analysis**

## **Exploratory Factor Analysis: Four Factor Solution for Men**

TITLE:

Single level EFA with four correlated continuous factors WLSMV estimate for

randomly selected male (Randsex group = 1)

DATA:

File = "~\SASCAT4.txt";

VARIABLE:

Names are

adult adultm adultw rand randsex arandsex memid hhid district block

Position Sex Age Education Occupation Marriage HHreligion HHcaste

HHwealthindex GMB GMBB CA DD DDB SSE SSEB SSF SSFB SSI SSIB TRUSTL

TRUSTN TRUSTS SCH SCB SCF householdmemberid respuniqueid psu;

Missing are all (-9999) ;

USEVARIABLES = GMBB CA DDB SSEB SSFB SSIB

TRUSTL TRUSTN TRUSTS SCH SCB SCF;

CATEGORICAL = GMBB CA DDB SSEB SSFB SSIB

TRUSTL TRUSTN TRUSTS SCH SCB SCF;

USEOBSERVATION ARE (randsex EQ 1);

ANALYSIS:

TYPE = EFA 4 4;

OUTPUT:

MODINDICES;

TECH1;

## **Confirmatory Factor Analysis: Four Factor Solution for Men**

TITLE:

Single level CFA with four correlated continuous factors WLSMV estimate for

randomly selected male (Randsex group = 3)

DATA:

File = "~\SASCAT4.txt";

VARIABLE:

Names are

adult adultm adultw rand randsex arandsex memid hhid district block

Position Sex Age Education Occupation Marriage HHreligion HHcaste

HHwealthindex GMB GMBB CA DD DDB SSE SSEB SSF SSFB SSI SSIB TRUSTL

TRUSTN TRUSTS SCH SCB SCF householdmemberid respuniqueid psu;

Missing are all (-9999) ;

USEVARIABLES = GMBB CA DDB SSEB SSFB SSIB

TRUSTL TRUSTN TRUSTS SCH SCB SCF;

CATEGORICAL = GMBB CA DDB SSEB SSFB SSIB

TRUSTL TRUSTN TRUSTS SCH SCB SCF;

USEOBSERVATION ARE (Sex EQ 3);

Analysis:

Estimator = WLSMV;

MODEL:

f1 BY GMBB* CA DDB;

f2 BY SSEB* SSFB SSIB;

f3 BY TRUSTL* TRUSTN TRUSTS;

f4 BY SCH* SCB SCF;

f1@1;

f2@1;

f3@1;

f4@1;

OUTPUT:

TECH1;

standardized;

SAVEDATA:

DIFFTEST IS aps_4FWLSMV_Male_V1_RG3.dat;

## **Exploratory Factor Analysis: Four Factor Solution for Women**

TITLE:

Single level EFA with four correlated continuous factors WLSMV estimate for

randomly selected women (Randsex group = 2)

DATA:

File = "~\SASCAT4.txt";

VARIABLE:

Names are

adult adultm adultw rand randsex arandsex memid hhid district block

Position Sex Age Education Occupation Marriage HHreligion HHcaste

HHwealthindex GMB GMBB CA DD DDB SSE SSEB SSF SSFB SSI SSIB TRUSTL

TRUSTN TRUSTS SCH SCB SCF householdmemberid respuniqueid psu;

Missing are all (-9999) ;

USEVARIABLES = GMBB CA DDB SSEB SSFB SSIB

TRUSTL TRUSTN TRUSTS SCH SCB SCF;

CATEGORICAL = GMBB CA DDB SSEB SSFB SSIB

TRUSTL TRUSTN TRUSTS SCH SCB SCF;

USEOBSERVATION ARE (randsex EQ 2);

ANALYSIS:

TYPE = EFA 4 4;

OUTPUT:

MODINDICES;

TECH1;

## **Confirmatory Factor Analysis: Four Factor Solution for Women**

TITLE:

Single level CFA with four correlated continuous factors WLSMV estimate for

randomly selected women (Randsex group = 4)

DATA:

File = "~\SASCAT4.txt";

VARIABLE:

Names are

adult adultm adultw rand randsex arandsex memid hhid district block

Position Sex Age Education Occupation Marriage HHreligion HHcaste

HHwealthindex GMB GMBB CA DD DDB SSE SSEB SSF SSFB SSI SSIB TRUSTL

TRUSTN TRUSTS SCH SCB SCF householdmemberid respuniqueid psu;

Missing are all (-9999) ;

USEVARIABLES = GMBB CA DDB SSEB SSFB SSIB

TRUSTL TRUSTN TRUSTS SCH SCB SCF;

CATEGORICAL = GMBB CA DDB SSEB SSFB SSIB

TRUSTL TRUSTN TRUSTS SCH SCB SCF;

USEOBSERVATION ARE (Sex EQ 4);

Analysis:

Estimator = WLSMV;

MODEL:

f1 BY GMBB* CA DDB;

f2 BY SSEB* SSFB SSIB;

f3 BY TRUSTL* TRUSTN TRUSTS;

f4 BY SCH* SCB SCF;

f1@1;

f2@1;

f3@1;

f4@1;

OUTPUT:

TECH1;

standardized;

SAVEDATA:

DIFFTEST IS aps_4FWLSMV_Female_V1_RG4.dat;

1. Beatty, P. C., & Willis, G. B. (2007). Research Synthesis: The Practice of Cognitive Interviewing. Public Opinion Quarterly, 71(2), 287–311. https://doi.org/10.1093/poq/nfm006 [↑](#footnote-ref-1)
